# Peroxins in Peroxisomal Receptor Export System Contribute to Development, Stress Response, and Virulence of Insect Pathogenic Fungus *Beauveria bassiana*

**DOI:** 10.3390/jof8060622

**Published:** 2022-06-10

**Authors:** Jia Hou, Haiyan Lin, Jinli Ding, Mingguang Feng, Shenghua Ying

**Affiliations:** Institute of Microbiology, College of Life Sciences, Zhejiang University, Hangzhou 310058, China; 12107004@zju.edu.cn (J.H.); 12007016@zju.edu.cn (H.L.); 0621538@zju.edu.cn (J.D.); mgfeng@zju.edu.cn (M.F.)

**Keywords:** peroxin, development, stress response, entomopathogenic fungus, development, virulence

## Abstract

In filamentous fungi, recycling of receptors responsible for protein targeting to peroxisomes depends on the receptor export system (RES), which consists of peroxins Pex1, Pex6, and Pex26. This study seeks to functionally characterize these peroxins in the entomopathogenic fungus *Beauveria bassiana*. BbPex1, BbPex6, and BbPex26 are associated with peroxisomes and interact with each other. The loss of these peroxins did not completely abolish the peroxisome biogenesis. Three peroxins were all absolutely required for PTS1 pathway; however, only BbPex6 and BbPex26 were required for protein translocation via PTS2 pathway. Three gene disruption mutants displayed the similar phenotypic defects in assimilation of nutrients (e.g., fatty acid, protein, and chitin), stress response (e.g., oxidative and osmotic stress), and virulence. Notably, all disruptant displayed significantly enhanced sensitivity to linoleic acid, a polyunsaturated fatty acid. This study reinforces the essential roles of the peroxisome in the lifecycle of entomopathogenic fungi and highlights peroxisomal roles in combating the host defense system.

## 1. Introduction

*Beauveria bassiana* is a prevalent causative fungus of mycosis in various arthropod species and has been wildly developed into various environment-friendly formulations for biological control of insect pests [1,2]. In the eco-system, infectious cells (e.g., conidia) adhere to the host cuticle and penetrate the host insect hemocoel with the help of extracellular hydrolytic enzymes. In the host hemocoel, the fungus undergoes a dimorphic change from mycelia into in vivo hyphal bodies (yeast-like cells) and rapid proliferation [3,4]. After killing the hosts, the fungus grows out of the insect hemocoel and develops into dense mycelia on the cadaver, which finally produces numerous conidia [5]. Peroxisomes are single membrane-bound organelles and generally involved in lipid metabolism and detoxification of reactive oxygen species (ROS) [6]. In filamentous fungi, peroxisomes have important roles in secondary metabolism [7], development [8], and pathogenicity [9]. Similarly, peroxisomes are involved in fatty-acid assimilation, stress response, development, and virulence in *B. bassiana* [10,11]. Our previous study showed the functional divergence of different peroxins and highlighted the essential roles of peroxisomes involved in biocontrol potential of the entomopathogenic fungi.

Currently, over 30 peroxins (PEX) have been implicated in peroxisome functionality such as biogenesis, proliferation, and the import of matrix proteins [12]. The import of matrix proteins from the cytoplasm into peroxisome relies on receptors Pex5 and Pex7 which are responsible for delivery of protein with peroxisomal targeting signal (PTS) type 1 and type 2, respectively [13]. In yeast, receptor export from the peroxisomal lumen depends on the docking complex consisting of Pex1, Pex6, and Pex15. In animals and filamentous fungi, the functional ortholog of yeast Pex15 is Pex26. Both Pex1 and Pex6 belong to AAA ATPase and are anchored to peroxisomes through interacting with the tail-anchored Pex15 or Pex26 [6,14]. In addition to their biochemical roles in receptor recycling, these components of the docking complex play various biological roles in filamentous fungi. In *Arthrobotrys* *oligospora* (a nematode-trapping fungus), Pex1 and Pex6 are involved in fatty-acid utilization, vegetative growth, conidiation, stress response, and pathogenicity [15]. In *Fusarium graminearum* (a plant pathogen), Pex1 plays an additional role in maintaining cell-wall integrity [16]. In another plant pathogen *Alternaria alternata*, Pex6 links peroxisomal biology to fungal conidiation, secondary metabolism, and pathogenicity [17]. In *Coniothyrium* *minitans* (a parasite of fungus), Pex6 is responsible for fatty-acid assimilation and oxidation resistance, which are involved in conidiation and parasitism [18]. Thus far, the roles of Pex26 in filamentous fungi have primarily been obtained from *Neurospora crassa*. In addition to recruiting the Pex1–Pex6 complex, Pex26 is enriched in the membrane of differentiated Woronin bodies, and its loss results in increased numbers of aberrantly small Woronin bodies [14]. Woronin bodies are unique membrane-bound organelles in filamentous fungi that quickly seal the septal pores when the hypha is damaged [6]. In *B. bassiana*, Woronin bodies are important for asexual development under aerobic and submerged conditions, which is critical for fungal virulence and potential as a biocontrol agent. The major protein of Woronin bodies (Hex1) is translocated into the peroxisome through the PTS1 pathway [11]. However, more investigations are still needed to reveal various functions of three components of the PTS receptor exporting complex in filamentous fungi.

In this study, we identified the homologs of three components of the PTS receptor exporting complex in *B. bassiana*, and their functions were genetically characterized. Our data revealed the architecture of the receptor recycling complex, as well as showed that their components exert physiological significance in *B. bassiana*.

## 2. Materials and Methods

### 2.1. Fungal Strains and Culturing Conditions

The wild-type *B. bassiana* ARSEF2860 (NCBI: txid655819) was used as a parent strain for constructing the gene disruption mutants and determining the subcellular localization of proteins. All fungal strains were maintained on Sabouraud dextrose agar (SDAY: 4% glucose, 1% peptone, 1.5% agar, and 1% yeast extract). *Escherichia coli* DH5α (Invitrogen, Carlsbad, CA, USA) for plasmid proliferation was cultured in Luria–Bertani (LB) medium with the necessary antibiotics. In fungal transformation, yeast extract broth (*w/v*: 0.5% sucrose, 1% protein, 0.1% yeast extract, 0.05% MgSO_4_, and 1.5% agar) was used to culture *Agrobacterium tumefaciens* AGL-1, which was used as a donor strain. Chemically defined medium Czapek–Dox agar (CZA) (3% sucrose, 0.3% NaNO_3_, 0.1% K_2_HPO_4_, 0.05% KCl, 0.05% MgSO_4_, and 0.001% FeSO_4_ plus 1.5% agar) was used in assays for screening transformant and phenotypic evaluation.

### 2.2. Bioinformatic Analyses of Three Pex Homologs in B. bassiana

The potential orthologs in the *B. bassiana* genome (NCBI: NZ_ADAH00000000.1) [19] were identified by BLAST search with corresponding query sequences which included *S. cerevisiae* Pex1 (GenBank No. P24004) and Pex6 (P33760), as well as *N. crassa* Pex26 (EAA28582). Domain analyses were performed through an online portal SMART (http://smart.embl-heidelberg.de/) (last accessed on 10 September 2021). The deduced protein sequences were used to analyze their phylogenetic relationship among the homologs from typical organism species. Protein sequences were downloaded from the NCBI database and aligned using CLUSTALW [20]. The phylogenetic tree was constructed using MEGA software (ver. 5), and the resultant tree from neighbor-joining analysis was evaluated with 1000 bootstrap replicates [21].

### 2.3. Targeted Gene Disruption and Complementation

All primers used in this study are given in Table S1. Gene disruption was conducted with the fluorescence-coupled double screening method. Complementation of gene loss was accomplished by introducing the whole gene into the gene disruption mutant strain [22]. The up- and downstream fragments of the indicated gene were amplified by primers Px1/Px2 and Px3/Px4 (X: Pex1, Pex6 and Pex26), respectively, and sequentially ligated into the *Xma*I/*Bam*HI sites and *Xba*I/*Spe*I sites in plasmid p0380-GTB with the phosphinothricin resistance gene (*bar*). The gene disruption vector was named p0380-X-KO and transformed into the wild-type strain. The transformants were screened on CZA plates with phosphinothricin (200 µg/mL) (45520, Sigma, St. Louis, MO, USA). The putative disruptants were screened by PCR reaction with primers Px5 and Px6, before further verification under a laser scanning confocal microscope. To complement the gene loss, the entire gene plus promoter, amplified with primers Px7 and Px8, was cloned into the vector p0380-sur-gateway with the chlorsulfuron resistance gene (*sur*), generating the plasmid p0380-sur-X. Transformants were grown on CZA plates with chlorsulfuron (C11325000, Dr. Ehrenstorfer GmbH, Augsburg, Germany) and screened by PCR with the primer pair Px5/Px6.

### 2.4. Assay for Fungal Vegetative Growth, Stress Response, Development, and Virulence

Phenotypic assays were conducted for the WT, gene disruption, and complemented mutant strains as described previously [11]. Each assay was repeated three times.

#### 2.4.1. Vegetative Growth

With a base of CZA medium, sucrose and NH_4_NO_3_ were replaced with carbon and nitrogen sources. Carbon sources (final concentration) included olive oil (0.3%), stearic acid (0.3%), palmitic acid (0.3%), oleic acid (0.3%), linoleic acid (0.002%), and sodium acetate (0.3%). Nitrogen sources (final concentration) included NaNO_3_ (0.3%), urea (0.5%), gelatin (0.5%), peptone (0.5%), and chitin (0.5%), using SDAY as the control nutrient-rich medium. The conidial suspension (1 µL, 10^6^ conidia/mL) was point-inoculated on plates. After an incubation of 7 days at 25 °C, the colony diameter was measured. SDAY was used as the control enriched medium.

#### 2.4.2. Stress Response

For chemical stress, aliquots of 1 μL suspensions (10^6^ conidia/mL) were inoculated onto the chemically supplemented CZA plates. Chemical reagents (final concentration) included 0.5 M NaCl, 1 M sorbitol, 2 mM H_2_O_2_, 0.02 mM menadione, and 3 μg/mL Congo red. Radial growth was measured after a 7 day incubation at 25 °C, using CZA plates without stress chemicals as a control.

To determine fungal response to linoleic acid (LA), two types of plates were used. On the basis of CZA, carbon and nitrogen sources were replaced with trehalose (3%) and peptone (0.5%), generating TPA plates. In CZA, LA concentration (*v/v*) were set as 0.002%, 0.005%, 0.01%, 0.02%, 0.04%, and 0.05%. In TPA, LA concentrations (*v/v*) were adjusted to 0.002%, 0.005%, 0.01%, 0.02%, 0.05%, and 0.1%. Culture medium without LA was used as a control.

#### 2.4.3. Development into Conidia and Blastospores

Under aerial conditions, aliquots of 100 µL conidial suspensions (10^7^ cells/mL) were inoculated on SDAY plates and incubated for 7 days at 25 °C. Mycelial discs were sampled, and the conidia on discs were quantified. Conidial production was shown as the conidial number per square centimeter. Under submerged conditions, conidia of the indicated strain were inoculated into SDB medium (SDAY without agar) at a final concentration of 10^6^ conidia/mL and cultured at 25 °C for 3 days with constant shaking (150 rpm) under darkness. The initial pH of SDB medium was 5.7. Blastospore concentration in the broth was quantified and shown as spore number per milliliter of broth.

#### 2.4.4. Insect Bioassay

Two infection methods were used to evaluate fungal pathogenicity against the final-instar larvae of *Galleria mellonella*, in which 35 larvae were grouped into one treatment. The indicated strain was cultured on SDAY plates, and the 7 day old conidia were used as inocula. In the cuticle infection assay, larvae were immersed in the conidial suspension (10^7^ conidia/mL) for 10 s. In the intrahemocoel infection assay, the conidial suspension (5 µL, 10^5^ cells/mL) was injected into the insect hemocoel. Tween-80 solution (0.02%) was used as a blank control. After inoculation, larvae were grown at 25 °C. The mortality was recorded every day, and the median lethal time (LT_50_) was calculated using the Kaplan–Meier method with a log-rank test. Mycoses on the cadaver were confirmed by incubating the cadaver in a moist chamber (saturated humidity) at 25 °C for 4 days with a photoperiod of 12 h/12 h (day/night).

### 2.5. Fluorescent Microcopy

Subcellular localizations of BbPex1, BbPex6, and BbPex26 were determined as described previously [11]. All primers are shown in Table S1. Firstly, gene mCherry was fused to the downstream of coding sequence for PTS2 targeting signal [11], and the hybrid gene was cloned into plasmid pBMRS [22]. This resultant vector was transformed into the wild-type strain, generating a transgenic strain with peroxisomes labeled with red fluorescence. The coding sequence for the indicated peroxin gene was amplified with primers PLx1 and PLx2 (X: Pex1, Pex6, and Pex26) using cDNA as the template. Resultant DNA fragment was ligated into the *Nco*I/*Eco*RI sites of pBMGB, in which the target gene was fused to the green fluorescent protein gene (GFP) [23]. The resulting plasmid was transformed into the above transgenic strain with peroxisomes labeled. The transformant was visualized on CZA plates with 200 μg/mL phosphinothricin. To view fluorescent signals, fungal strains were cultured in SDB medium (SDAY plate without agar) at 25 °C for 2 days. The resultant mycelia were sampled, and the fluorescent signals were examined under a laser scanning confocal microscope (LSM 710, Carl Zeiss Microscopy GmbH, Jena, Germany).

The translocation process of peroxisomal proteins was examined as described previously [11]. Expression plasmid pBMS-GFP-PTS1 [24] generated protein GFP with PTS1 at the C-terminus, while plasmid pBMGS-BbThi-GFP [11] generated protein GFP with PTS2 at the N-terminus. The resultant plasmid was introduced into the wild-type, Δ*Bbpex1*, Δ*Bbpex6*, and Δ*Bbpex26* mutant strains. To examine fluorescent signals in mycelia, the indicated strain was cultured in SDB medium for 2 days at 25 °C. Resultant mycelia were rinsed with water and examined under a confocal laser scanning microscope.

### 2.6. Imaging Peroxisomes with Transmission Electron Microscopy

Peroxisomal morphology was examined using transmission electron microscopy (TEM) as described previously [25]. Fungal strains were grown in SDB at 25 °C for 2 days. Resultant mycelia were washed and fixed in K_2_MnO_4_ solution (*w/v*: 1.5%) for 20 min at 20 °C. The fixed samples were dehydrated and embedded in resin. Ultrathin sections were stained and examined under a transmission electron microscope (Model H-7650, Hitachi).

### 2.7. Yeast Two-Hybrid (Y2H) Analyses

Yeast two-hybrid (Y2H) assays were used to determine the direct interaction between paired proteins using a Matchmaker™ GAL4 Two-Hybrid System 3 Kit (Clontech Laboratories, Mountain View, CA, USA) [26]. All primers are listed in Table S1. In brief, BbPEX6 and BbPEX26 were amplified and cloned into the vector pGBKT7, whereas *BbPEX1* and *BbPEX6* were cloned into the vector pGADT7. Paired plasmids were transformed into yeast strain YH109, and the resultant transformant was screened on SD/Leu–Trp medium. The positive interaction was determined when the transformants grew well on SD/Trp–Leu–His–Ade plates. Strains for positive and negative control were provided by the kit.

### 2.8. Statistical Analyses

One- and two-way analyses of variance (ANOVA) were applied in statistical analysis of phenotypic measurements for all strains, and the significance was determined by Tukey’s honestly significant difference test (Tukey’s HSD). Prior to ANOVA, all data were checked with Bartlett’s test for homoscedasticity.

## 3. Results

### 3.1. Bioinformatic Characterization and Molecular Manipulation

On the basis of the BLAST search, the *B. bassiana* homologs of yeast Pex1 and Pex6 were identified as BBA_01173 (BbPex1) and BBA_07046 (BbPex6), respectively. The *B. bassiana* homologs of *N. crassa* Pex26 were characterized as BBA_ 01058 (BbPex26). The ORF sequence of BbPex1 was 3825 bp long with two introns in genome; the BbPex6 ORF was 4304 bp long with two introns, while the BbPex26 ORF was 1428 bp long with one intron. BbPex1 consisted of multiple domains, including one PEX-1N (PF09262.11), one AAA_lid_3 (AAA_lid_3) and two AAA (PF00004.29) domains. BbPex6 contained two AAA (PF00004.29) domains. However, only a Pex26 domain (PF07163) was revealed in BbPex26. Phylogenetic analyses indicated that BbPex1 and BbPex6 had a close relationship with their respective homologs in *S. cerevisiae*. BbPex26 had a relatively closer relationship with its homologs in fungal species than those in mammals, although all Pex26 homologs were sorted into one branch. In addition, Pex15 (*S. cerevisiae*) and Pex26 (mammals and filamentous fungi) were grouped into different branches (Figure S1).

**Figure 1:**
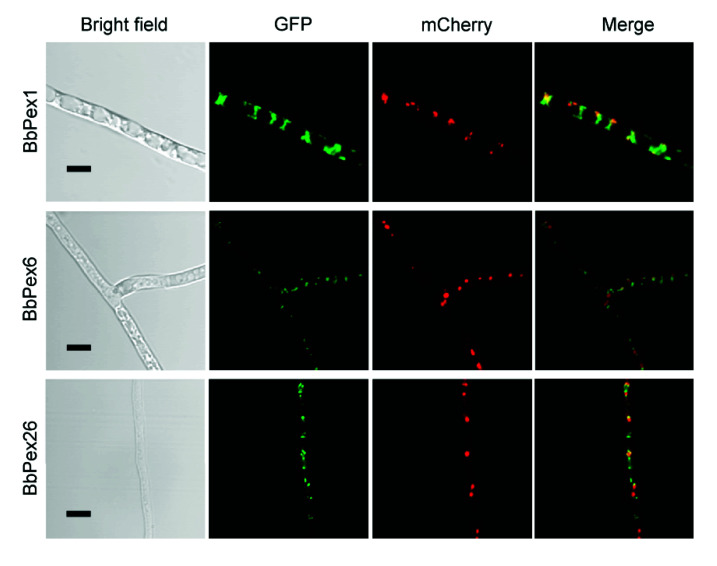
Subcellular analyses of three peroxins in *B. bassiana*. The coding sequence of the indicated peroxin was fused to the green fluorescent protein gene (*GFP*), and peroxisomal targeting signal sequences were fused to mCherry gene. Two resultant hybrid genes were transformed into the wild-type strain. Mycelia were cultured in SDB medium, and fluorescent signals were examined under a laser scanning confocal microscope. Scale bars: 5 µm.

To view the subcellular localization of three peroxins, their coding sequences were fused to a green fluorescent protein gene. Peroxisomes were indicated by fusing the PTS2 sequence to the mCherry gene. As shown in Figure 1, in all transgenic *B. bassiana* strains expressing the fused gene, globular green signals were colocalized with the red fluorescence well. These results indicated that the three peroxins were localized at the peroxisomes.

Protein interactions were tested through an Y2H system (Figure S2). BbPex1 and BbPex6 were used as prey, while BbPex6 and BbPex26 were used as bait. All paired combinations gave yeast transformants the ability to grow on SD/Leu–Trp–His–Ade medium. This result indicated that the three peroxins interacted with each other.

To determine the physiological functions of the three peroxins, their single disruption mutants were constructed with a strategy of target replacement. To restore the gene loss, the entire ORF of the indicated gene with its promoter was ectopically introduced into the genome of the indicated disruptants. All gene disruption strains were successfully verified using a PCR reaction and fluorescence assay (Figure S3).

### 3.2. Imaging the Peroxisome Biogenesis and the Translocation of the Peroxisomal Proteins

**Figure 2:**
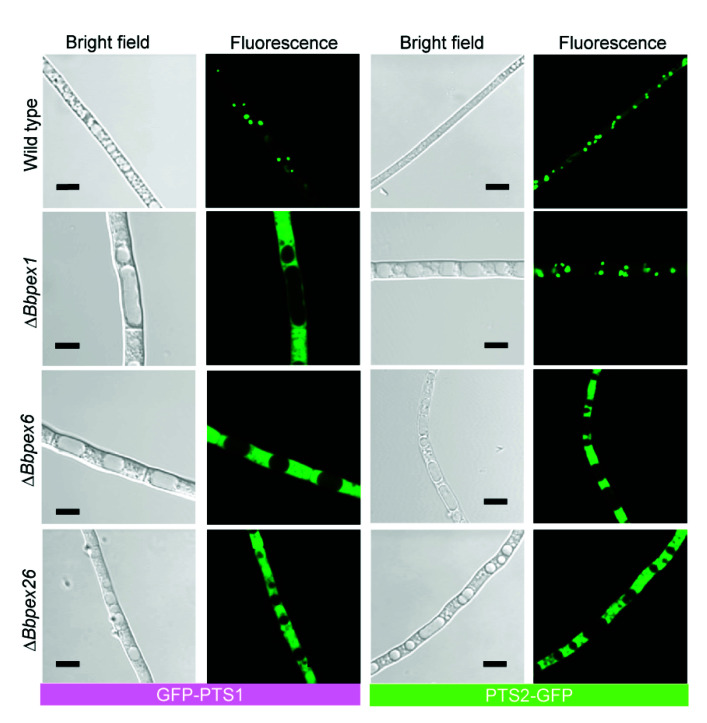
Translocation processes of peroxisomal proteins. Peroxisomal targeting signal (PTS) type 1 and type 2 were fused to the green fluorescent protein gene (GFP). The fusion gene was introduced into the wild-type and Δ*Bbpex1*, Δ*Bbpex6*, and Δ*Bbpex26* mutant strains. Granular signals were obviously observed in the wild-type strain. Signals from GFP-PTS1 were evenly distributed in the cytoplasm. As for PTS2-GFP, granular signals were only observed in the Δ*Bbpex1* strain. Scale bar: 5 µm.

As shown in Figure S4, peroxisomes were observed in the wild-type and three gene disruption strains (Δ*Bbpex1*, Δ*Bbpex6*, and Δ*Bbpex26*), although the peroxisomal number was decreased in all disruptants. No significant difference was noted for peroxisomes in the three disruptants when compared with the wild-type strains. This result indicated that gene disruption did not completely block the formation of peroxisomes. GFP-SKL and BbThi-GFP were used as reporters to view the activities of PTS1 and PTS2 pathways, respectively. The indicator gene was individually transformed into the wild type and three gene disruption mutants. As shown in Figure 2 (left panel), granular green signals were obviously seen in the wild-type strain, but the GFP signals were evenly distributed in cytoplasm of three gene disruption mutants. As shown in Figure 2 (right panel), granular green signals were obviously observed in the wild-type and Δ*Bbpex1* mutant strains, but the GFP signals were evenly distributed in the cytoplasm of the other two disruptants. Together, these results indicated that the loss of these three peroxins did not affect peroxisome biogenesis, but had a distinct impact on the translocation of peroxisomal proteins.

### 3.3. Phenotypic Assays for Vegetative Growth and Development

**Figure 3:**
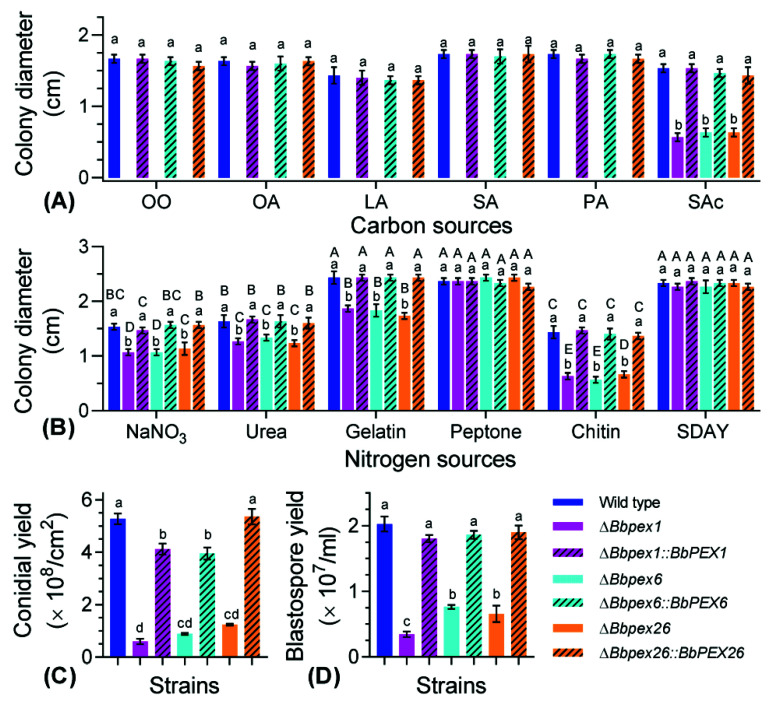
Fungal vegetative growth and development. Considering the minerals in CZA, various carbon (**A**) and nitrogen (**B**) sources were assayed. Carbon sources included olive oil (OO), oleic acid (OA), linoleic acid (LA), stearic acid (SA), palmitic acid (PA), and sodium acetate (SAc). SDAY was used as the control nutrient-rich medium. After a 7-d incubation at 25 °C, the colony diameter was examined. (**C**) Conidial production on plates. Fungal strains were inoculated on SDAY plates and cultured at 25 °C. The conidial yield was examined at 8 days post incubation. (**D**) Blastospore formation under submerged conditions. Fungal strains were cultured in SDB at 25 °C with aeration. Blastospore yield was quantified at 3 days post incubation. The lowercase letters indicate a significant difference in the indicated phenotype among different strains, while the uppercase letters indicate a significant difference among different nitrogen sources in the indicated strain (Tukey’s HSD: *p* < 0.05). Error bars: standard deviation.

Vegetative growth was examined on different nutrients. As for carbon sources (Figure 3A), colony diameters differed significantly among the seven strains on the indicated nutrient. Compared with the wild-type strain, severe growth defects of three disruptants were observed on all tested carbon sources; in particular, the three disruptants did not form an obvious colony on five lipids (olive oil, oleic acid, linoleic acid, stearic acid, and palmitic acid). On sodium acetate, colony diameters significantly differed among the wild-type and three disruptants (*F*_6, 14_ = 140.6, *p* < 0.01). No significant difference in colony diameter was observed among the wild-type and three complementation mutant strains on all tested carbon sources. As for nitrogen sources (Figure 3B), colony diameters differed significantly among the seven strains (*F*_6, 84_ = 127.8, *p* < 0.01) and six nutrients (*F*_5, 84_ = 1223.7, *p* < 0.01). On peptone and SDAY plates, no significant difference in colony diameter was observed among all tested strains. On the chitin plates, colony diameters for three disruption mutants ranged from 0.57 to 0.67 cm, with an approximate 60% reduction when compared with the wild-type strain (1.43 ± 0.12 cm) (mean ± standard deviation (SD)).

All disruptants displayed impaired ability in sporulation. As for conidiation on SDAY plates (Figure 3C), a significant difference in conidial production was observed among the seven strains (*F*_6, 14_ = 386.7, *p* < 0.01). Conidial yield of the wild-type strain was 5.27 ± 0.20 × 10^8^ conidia/cm^2^ (mean ± SD), no complementation strains displayed a significant difference with the wild-type strain. When compared with the wild-type strain, Δ*Bbpex1,* Δ*Bbpex6*, and Δ*Bbpex26* mutants displayed reductions of approximately 88%, 83%, and 76% in conidial production, respectively. Under submerged conditions (Figure 3D), the wild-type strain produced 2.03 ± 0.11 × 10^8^ spores/mL. Blastospore production was significantly reduced in the three gene disruption mutants, with approximate reductions of 93%, 85%, and 87%, respectively, when compared with those of the wild-type and complementation mutant strains (*F*_6, 14_ = 218.1, *p* < 0.01). In addition, ablation of the three peroxins exerted different effects on spore formation, with the loss of BbPex1 causing a more significant effect.

### 3.4. Fungal Response to Stresses

**Figure 4:**
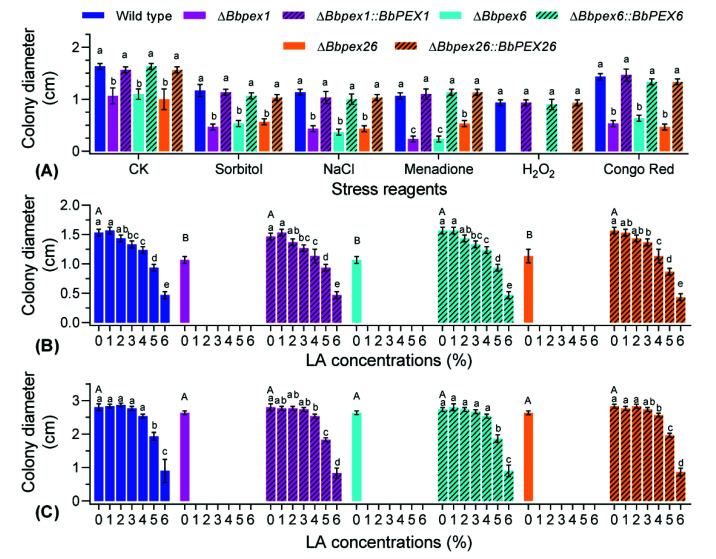
Vegetative growth under chemical stress. (**A**) Stress chemicals were included in the CZA to generate various stresses. CZA without stress reagent was used as a control (CK). Numbers (0–6) indicate the gradient concentrations of LA. (**B**) In CZA, LA concentrations (*v/v*) were set as 0%, 0.002%, 0.005%, 0.01%, 0.02%, 0.04%, and 0.05%. (**C**) In TPA, LA concentrations were set as 0%, 0.002%, 0.005%, 0.01%, 0.02%, 0.05%, and 0.1%. The conidial suspension was inoculated onto plates and cultured at 25 °C. Seven days later, the colony diameter was examined. The lowercase letters indicate a significant difference in the indicated phenotype among different strains, while the uppercase letters indicate a significant difference in colony diameter among different strains under control condition (Tukey’s HSD: *p* < 0.05). Error bars: standard deviation.

Chemical stress was generated by integrating the indicated chemical reagent (Figure 4A). Colony diameters differed significantly among the seven strains under the indicated stress. On the control medium (CZA), gene loss resulted in a slight decrease in colony diameter, with a reduction from 33% to 38%. Under all stressful conditions, the reduction in colony diameter was not less than 50% (Bbpex26 under menadione stress). Strikingly, all disruption mutants were particularly sensitive to oxidative stress caused by hydrogen peroxide and did not form an obvious colony.

Fungal resistance to LA stress was evaluated on two types of medium. On CZA plates (Figure 4B), colony diameters differed significantly among the seven strains at different concentrations. Under LA stress, the colony diameter of the wild-type strain decreased with the LA concentrations with a critical concentration of 0.05%. No disruptants produced a visible colony even on the plates with 0.002% LA. In addition, the varying trends for complementation mutants did not significantly differ with that for the wild-type strain. On TPA plates (Figure 4C), a significant difference in colony diameter was detected among the seven strains at different concentrations. The colony diameters of the wild-type strain were not significantly different for the concentrations of 0–0.01% and decreased gradually with increasing LA concentrations from 0.02%. These results indicated that the loss of the three peroxin genes resulted in enhanced sensitivity to LA stress, which varied with ambient nutritional conditions.

### 3.5. Fungal Virulence against Insects

**Figure 5:**
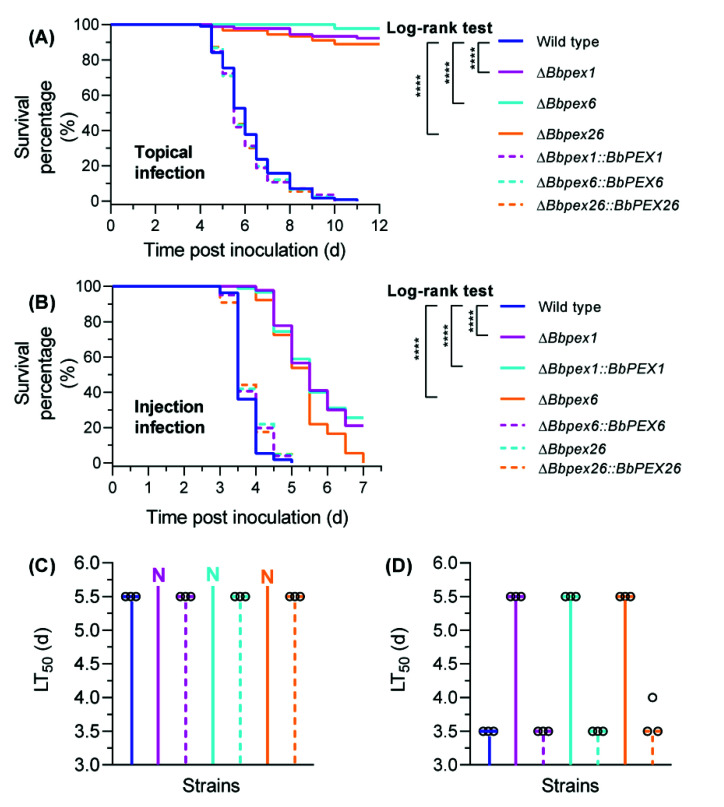
Fungal virulence against the insect host. In the topical infection assay (**A**), the hosts were immersed in the conidial suspension (10^7^ conidia/mL). In the intrahemocoel injection assay (**B**), the conidial suspension (5 µL, 10^5^ conidia/mL) was injected into the host hemocoel. The infected hosts were reared at 25 °C. The survival percentage was recorded and used to calculate the median lethal time (LT_50_) for the topical infection (**C**) and intrahemocoel injection assays (**D**) using Kaplan–Meier analyses. The hollow circles indicate the observed values for LT_50_. ‘N’ indicates that the LT_50_ value could not be calculated due to an accumulative mortality less than 50%. A log-rank test was used to determine statistical differences between the paired curves. ****: *p* < 0.0001.

In the topical infection bioassay (Figure 5A), mortality was recorded over a 12 day period. No death was observed in the control group treated with Tween-80 solution. The wild-type and all complementation mutant strains killed all bioassay insects. Survival percentages for Δ*Bbpex1,* Δ*Bbpex6*, and Δ*Bbpex26* mutants were 92.2%, 97.8%, and 88.9%, respectively. As shown in Figure 5C, the LT_50_ for the wild-type strain was 5.70 ± 0.43 days, and no complemented strains showed a significant difference with the wild-type strain. The LT_50_ values for gene disruption mutants could not be estimated owing to their mortalities being less than 50%. In intrahemocoel infection assays, mortality was recorded for 6.5 days (Figure 5B).The wild-type and all complementation mutant strains resulted in a cumulative mortality of 100%. The LT_50_ values for Δ*Bbpex1,* Δ*Bbpex6* and Δ*Bbpex26* mutants were 5.44 ± 0.04, 5.48 ± 0.11, and 5.01 ± 0.12 days, respectively, whereas that for the wild-type strain was 3.45 ± 0.09 days (Figure 5D). No significant difference in LT_50_ value was observed between the wild-type and each complemented strain. In the host hemocoel, no disruptants produced detectable fungal cells (Figure S5A). These results indicated that the loss of these peroxin genes significantly weakened the fungal virulence, particularly when infecting hosts via cuticle.

After being kept in moist conditions for 4 days, no gene disruption mutants produced obvious mycelia on the cadavers, whereas the wild-type and complemented strains produced thick mycelia (Figure S5B).

## 4. Discussion

Peroxisomes are generally conserved in eukaryotic organisms. Peroxisomal proteins are synthesized in the cytoplasm and imported into the organelles, in which receptors are responsible for the recognition of proteins with PTS1 and PTS2 signals. In animals and filamentous fungi, the process of receptor export from peroxisomes depends on the complex consisting of Pex1, Pex6, and Pex26 [6,14]. In this study, the orthologs of Pex1, Pex6, and Pex26 were also present in *B. bassiana*, a well-known entomopathogenic fungus. In *B. bassiana*, pex1 and pex6 are both AAA ATPases, and Bbpex26 only contains a Pex26 domain. This indicates that the domain organization of these three peroxins is conserved among the orthologs in other organisms [6]. Functional analyses revealed that these three peroxins contribute to fungal development, stress response, and virulence in *B. bassiana*.

As expected, these three peroxins were localized in peroxisomes, but dispensable for the biogenesis of peroxisomes, which is similar to the roles of Pex5 and Pex7 in *B. bassiana* [11]. However, ablation of Pex1 and Pex6 resulted in almost no detectable peroxisomes in *A. oligospora* [15]. In Chinese hamster ovary cells, Pex26 contributes to formation of peroxisomes [27]. These findings suggest that *B. bassiana* has a different mechanism involved in the biogenesis of peroxisomes. The direct interaction between Pex1 and Pex6 plays a critical role in peroxisome biology [28,29]. The tail-anchored Pex15 or Pex26 recruits the Pex1–Pex6 complex onto peroxisomes [30]. In *B. bassiana*, these three peroxins interact with each other. This result suggests that the Pex1–Pex6 interaction is conserved in *B. bassiana*, and the anchor protein has potential binding activity with any component in the ATPase complex. However, in mammals, Pex26 interplays with the Pex1–Pex6 complex in a Pex6-dependent manner [27]. In addition, Pex1, Pex6, and Pex26 are all required for translocation activity of the PTS1 pathway, but Pex1 is not required for the activity of PTS2 pathways in *B. bassiana*. BbPex5 and BbPex7 independently control the PTS1 and PTS2 pathways in peroxisomal protein import, respectively [11]. These findings suggest that *B. bassiana* adopts a simpler complex for exporting Pex7 from the peroxisome to the cytoplasm.

Unlike insect pathogenic bacteria and viruses, *B. bassiana* infects the host via penetrating the host cuticle. Conidial germination and further growth are essential for infection initiation [31]. The insect cuticle consists of complex mixtures of protein, chitin, lipid, alkane, etc. [32]. *B. bassiana* penetrates the host cuticle from outside to inside using a plethora of nutrients in the cuticle [33]. To assimilate alkane in the host cuticle, *B. bassiana* activates peroxisome proliferation and the expression of several genes associated with oxidative stress and peroxisome biogenesis [34]. The peroxisome plays a more important role in fungal virulence in the cuticle infection route than in the direct intrahemocoel infection. These findings suggest that the peroxisome is essential for fungal assimilation of nutrients and response to oxidative stress at the early stage of infection. In *B. bassiana*, Pex1, Pex6, and Pex26 are all required for assimilation of the above macromolecules, and they are particularly indispensable for fatty-acid utilization. In fungi, fatty acids are mainly degraded in peroxisomes. Pex1 and Pex6 contribute to the conserved role of peroxisomes in fatty-acid degradation [15,18]. However, BbPex5 is necessary for fatty-acid assimilation, but BbPex7 is not [11]. These results reinforce that the receptor export complex is indispensable for the activity of the PTS1 pathway. Ablation of peroxin genes resulted in different radial growth on different carbon and nitrogen sources. We deduce that *B. bassiana* uses different pathways to use carbon and nitrogen sources, among which some pathways are localized in the peroxisomes. In *Ustilago maydis* (a phytopathogenic fungus), glycolytic enzymes are localized in the peroxisomes. Any damage to the peroxisome resulted in the impaired effects on glycolysis [35]. Future studies are needed to decipher the peroxisomal pathways involved in primary metabolism.

After invading the host hemocoel, *B. bassiana* develops into hyphal bodies (*in vivo* blastospore) via a process of dimorphic change [3,36]. BbPex5 and BbPex7 have only a slight contribution to fungal development into the blastospore [11]. The current study revealed that the three peroxins in the receptor export complex have a more significant contribution to fungal dimorphism. This implies that the receptor export complex regulates additional pathways in dimorphism.

Fungal resistance to stress is critical for the potential virulence of the fungus as a biocontrol agent [31]. *B. bassiana* encounters reactive oxygen species (ROS) (e.g., O^2−^) when interacting with the host immune defense [37]. The three peroxins in the receptor export complex significantly participate in fungal resistance to oxidative stress, which is identical to the roles of BbPex5 and BbPex7 [11]. Similar results were also observed in other filamentous fungi (e.g., *A. oligospora* and *F. graminearum*) [15,16]. In *Metarhizium* *robertsii* (a sister entomopathogenic fungus), peroxisomal activity and peroxin genes are involved in fungal responses to oxidative stress [38]. Under submerged conditions, Woronin bodies are involved in peroxisome biogenesis and the stress response in *B. bassiana* during microsclerotia formation [10]. In insect hemocoel, hemolymph causes osmotic stress on fungal cells [39]. The above three peroxins are involved in fungal response to osmotic and cell-wall stress, whereas BbPex5 and BbPex7 are not [11]. In addition, the three peroxins mediate fungal tolerance to toxicity caused by polyunsaturated fatty acids (e.g., linoleic acid). In mammal cells, peroxisome deficiency causes dysfunction of fatty-acid oxidization, which produces lipotoxic fatty acids (e.g., palmitic acid and linoleic acid) [40]. This suggests that the fatty-acid oxidization in the peroxisome not only supplies cells with energy, but also maintains the cellular homeostasis of free fatty acids assumed to cause cytotoxicity in excess. Insects accumulate various fatty acids in the cuticle and hemolymph, which are involved in immune defense against fungal pathogens [32,41]. Collectively, the three peroxins significantly contribute to fungal virulence owing to their roles in nutrient assimilation, dimorphism, and stress resistance.

After killing the hosts, *B. bassiana* grows out the cadaver and develops into intensive mycelia followed by conidiation [42]. On nutrient plates, three peroxin genes significantly contribute to conidiation. This is a well-known role of the three genes in filamentous fungi, as established in *A. oligospora*, *C. minitan*, and *F. graminearum* [15,16,18]. A previous study unraveled that BbPex5 and BbPex7 are also essential for conidiation [11]. These results stress that the ‘core’ peroxins maintain the peroxisomal functionality during fungal differentiation. Evidently, *B. bassiana* only reaches the cadaver surface when it penetrates through the host cuticle from the body cavity. The three peroxins in the receptor export complex significantly function in fungal translocation in the cadaver from inside to outside. The host cuticle contains the fabricated chitin layer [33]. This result can be explained by the significant role of the receptor export complex in the degradation of macromolecules, particularly chitin. These findings suggest that peroxisomes are critical for the ability of the fungus to penetrate the host cuticle by mediating chitin assimilation, although the detailed mechanisms deserve further investigation. In the natural environment, the conidiation process produces numerous conidia, which promote fungal dispersal and survival [43,44]. As for *B. bassiana*, conidia on the cadaver function as initial inocula for subsequent infection cycles [41]. In this sense, the peroxisome plays a determinant role in maintaining the infection cycle.

## 5. Conclusions

In summary, BbPex1, BbPex6, and BbPex26 are associated with peroxisomes and interact with each other. These three peroxins are indispensable for the PTS1 pathway; however, only BbPex6 and BbPex26 are required for the activity of the PTS2 pathway. As for biological functions, these three peroxins significantly contribute to fungal development, stress tolerance, and virulence. Notably, the components of the receptor export complex link the peroxisomal function with fungal resistance to stress caused by polyunsaturated fatty acids. This study highlights the essential roles of peroxisomes in the lifecycle of entomopathogenic fungi, particularly in combating the host immune defense.

## Data Availability

Not applicable.

## Supplementary Materials

The following supporting information can be downloaded at https://www.mdpi.com/article/10.3390/jof8060622/s1. Figure S1. Bioinformatic characterization of Pex1, Pex6, and Pex26 in *B. bassiana*; Figure S2. Protein interaction test in yeast two-hybrid (Y2H) system; Figure S3. Construction of the gene disruption and complemented mutant strains; Figure S4. Ultrastructure for mycelia of *B. bassiana*; Figure S5. Pathogenic and saprotrophic growth of *B. bassiana*; Table S1. Primers used in this study.
