# Supplementary material for: Peroxins in Peroxisomal Receptor Export System Contribute to Development, Stress Response, and Virulence of Insect Pathogenic Fungus Beauveria bassiana
Source: J Fungi (Basel). 2022 Jun 10;8(6):622. doi: 10.3390/jof8060622 (PMC9224678; doi:10.3390/jof8060622)

Figure S1 Bioinformatic characterization of Pex1, Pex6, and Pex26 in *B. bassiana*. Phylogenetic relationship was constructed with Neighbor joining analysis and the bootstrap values from 1000 replicates are shown as numbers at each branch, using Pex5 and Pex7 as reference. Abbreviations for organism species were followed by GenBank accession numbers of their respective gene. An: *Aspergillus nidulans*; At: *Arabidopsis thaliana*; Bb: *Beauveria bassiana*, Hs: *Homo sapiens*; Mm: *Mus musculus*; Sc: *Saccharomyces cerevisae*; Yl: *Yarrowia lipolytica*.

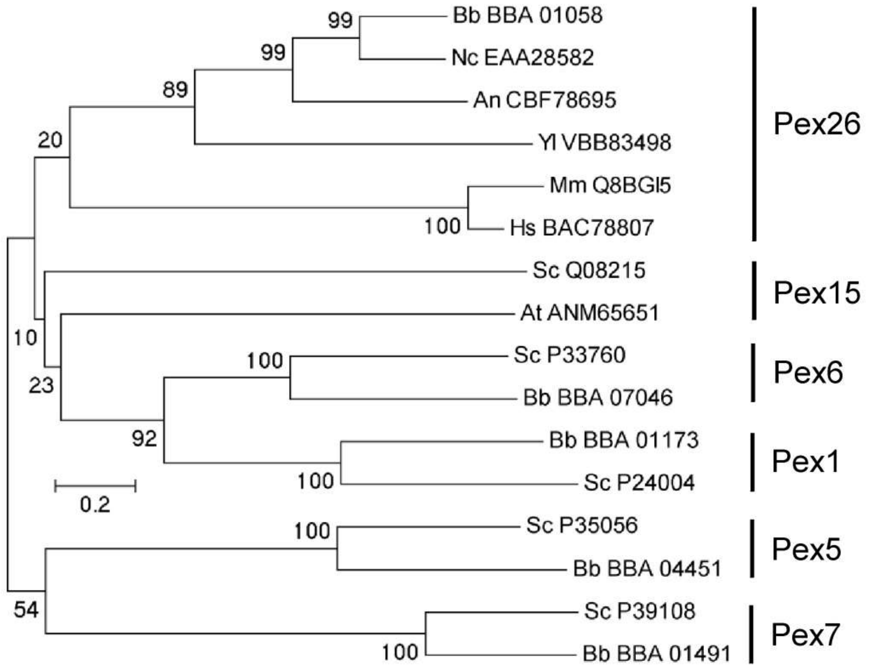

Supplement: Supplementary file 1 [file jof-08-00622-s001.zip › Figure S1.pdf]
