# Supplementary material for: Peroxins in Peroxisomal Receptor Export System Contribute to Development, Stress Response, and Virulence of Insect Pathogenic Fungus Beauveria bassiana
Source: J Fungi (Basel). 2022 Jun 10;8(6):622. doi: 10.3390/jof8060622 (PMC9224678; doi:10.3390/jof8060622)

Figure S2 Protein interaction test in yeast two-hybrid (Y2H) system. *BbPEX6* and *BbPEX26* were cloned into the vector pGBKT7 (AD); whereas, *BbPEX1* and *BbPEX6* were cloned into the vector pGADT7 (BD). Paired plasmids were transformed into yeast strain YH109 and resultant transformant was screened on SD/Leu-Trp (SDTL) medium. The positive interaction was determined when the transformants grew well on SD/Trp-Leu-His-Ade (SD-TLHA) medium. The control strains were provided by kit.

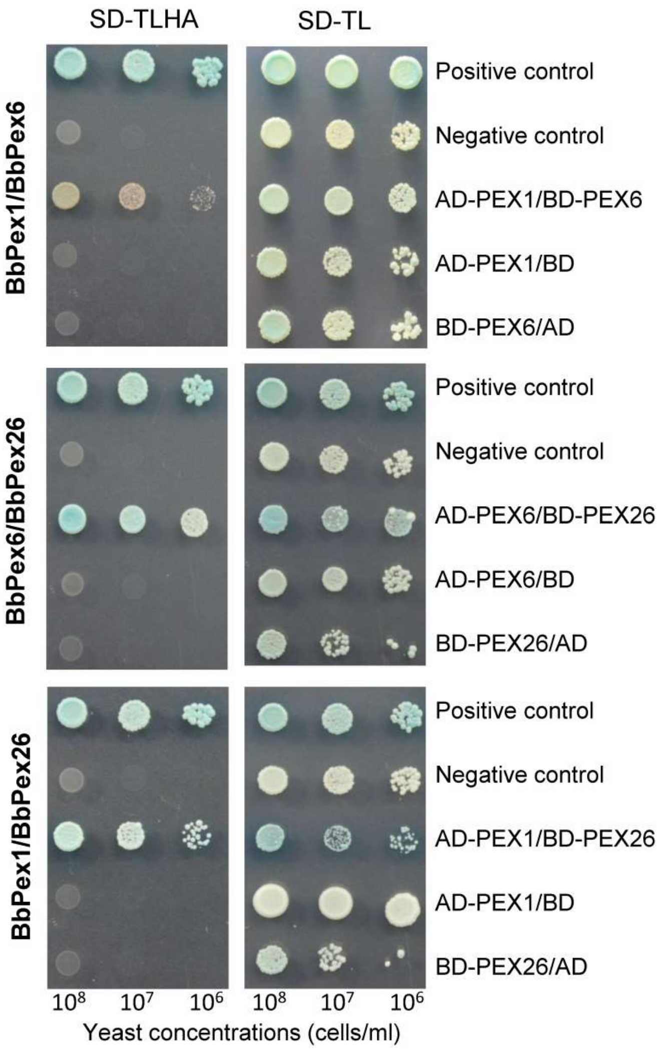

Supplement: Supplementary file 1 [file jof-08-00622-s001.zip › Figure S2.pdf]
