# Supplementary material for: Peroxins in Peroxisomal Receptor Export System Contribute to Development, Stress Response, and Virulence of Insect Pathogenic Fungus Beauveria bassiana
Source: J Fungi (Basel). 2022 Jun 10;8(6):622. doi: 10.3390/jof8060622 (PMC9224678; doi:10.3390/jof8060622)

Figure S3 Construction of the gene disruption and complemented mutant strains. (A) PCR screening for the gene disruption and complemented mutants. Lane1: wild type, lane 2:disruption mutant, lane 3: complemented strain. DNA marker was used indicate the fragment size. (B) Representative images for homologous (HR) and non-homologous recombination (NHR) events. The fluorescent signals were detected under a laser scanning confocal microscope. The wild-type strain is used as negative control. Bars: 25 μm.

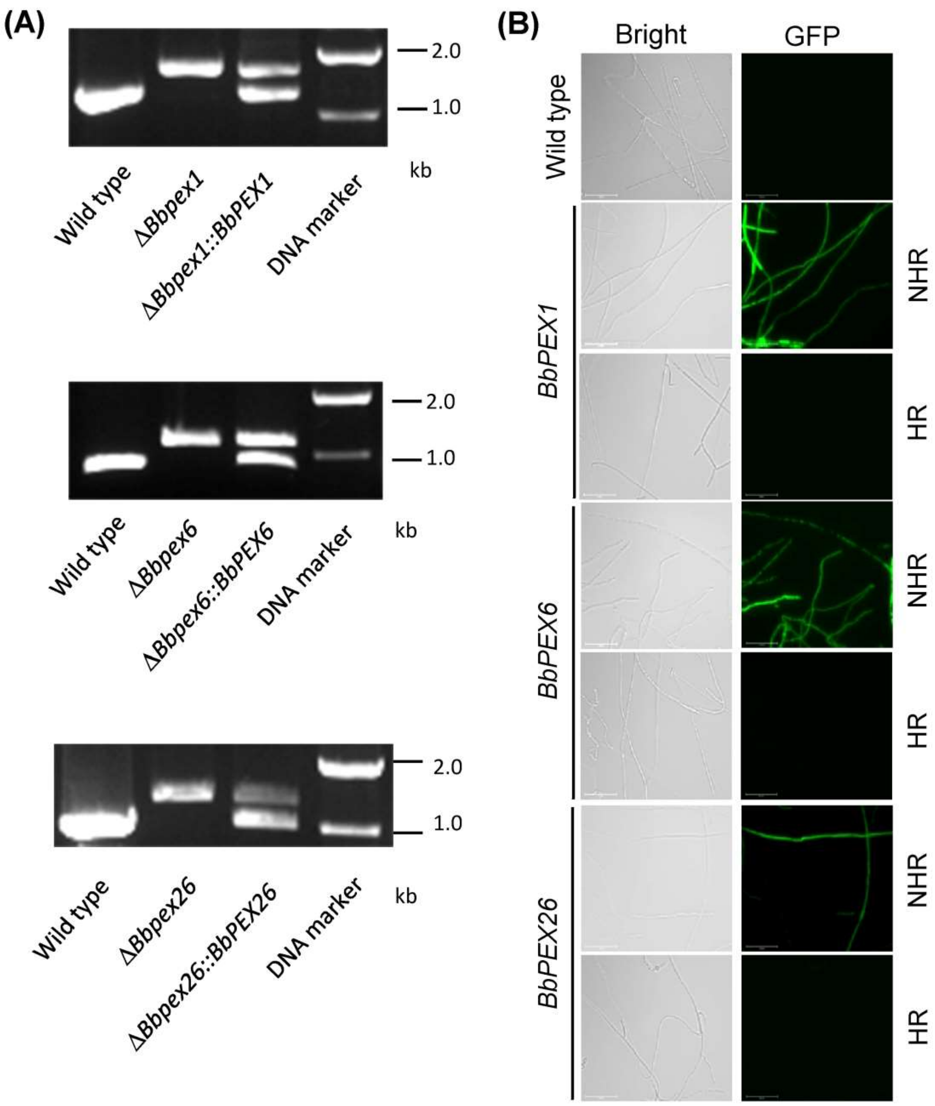

Supplement: Supplementary file 1 [file jof-08-00622-s001.zip › Figure S3.pdf]
