# Supplementary material for: Peroxins in Peroxisomal Receptor Export System Contribute to Development, Stress Response, and Virulence of Insect Pathogenic Fungus Beauveria bassiana
Source: J Fungi (Basel). 2022 Jun 10;8(6):622. doi: 10.3390/jof8060622 (PMC9224678; doi:10.3390/jof8060622)

Figure S4 Ultra-structure for mycelia of *B. bassiana*. Transmission electron microscopy was used to examine mycelial ultra-structures. There was no significance in peroxisome formation between the wild-type and each gene disruption mutant strain.

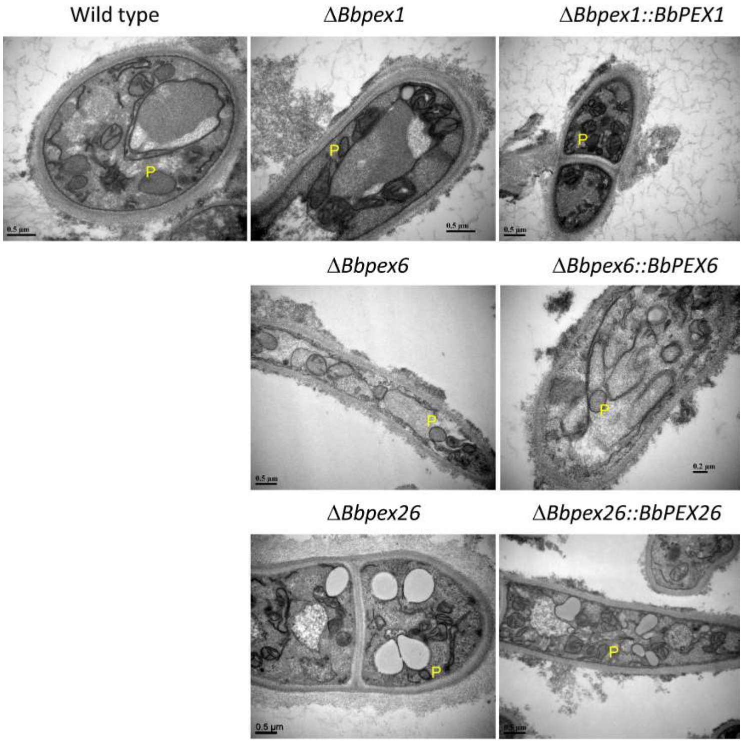

Supplement: Supplementary file 1 [file jof-08-00622-s001.zip › Figure S4.pdf]
