# Supplementary material for: Peroxins in Peroxisomal Receptor Export System Contribute to Development, Stress Response, and Virulence of Insect Pathogenic Fungus Beauveria bassiana
Source: J Fungi (Basel). 2022 Jun 10;8(6):622. doi: 10.3390/jof8060622 (PMC9224678; doi:10.3390/jof8060622)

Figure S5 Pathogenic and saprotrophic growth of *B. bassiana*. (A) Imaging for *in vivo* hyphal bodies and mycelia. Conidia suspension was injected into the host hemocoel, and the hosts were incubated for 3.5 d at 25℃. Fungal cells in hemolymph were sampled and examined under microscope. Scale bars: 20 μm. HC: hemocyte, FC: fungal cell. (B) Mycoses on cadavers. Cadavers were incubated in a moist box for 4 d at 25℃. Scale bars: 1 cm.

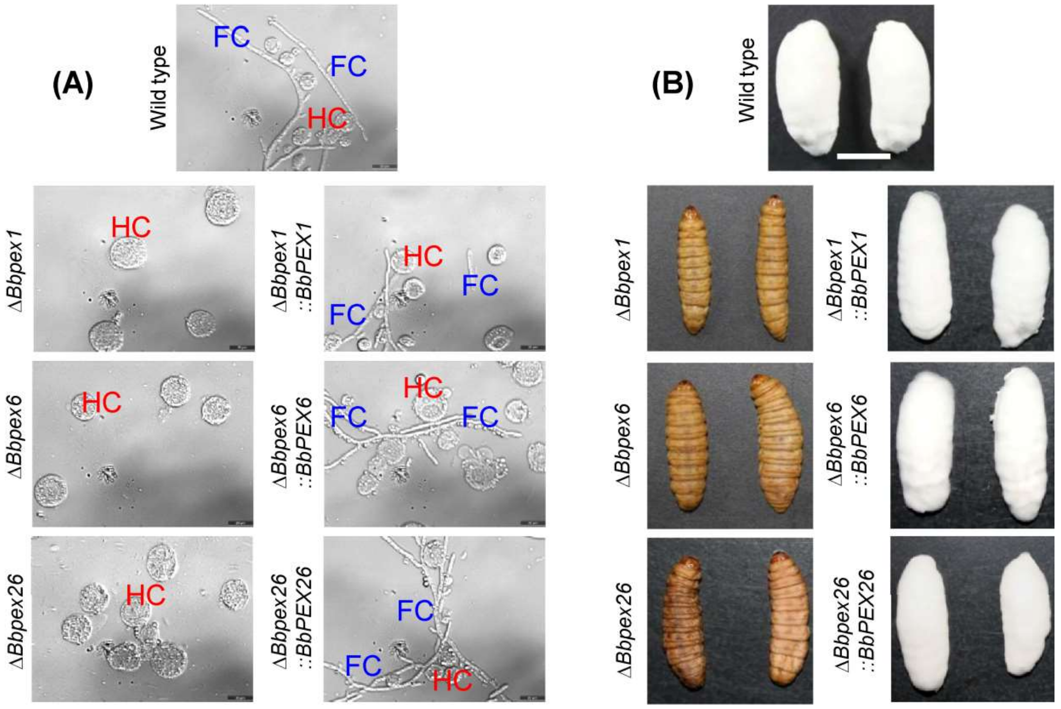

Supplement: Supplementary file 1 [file jof-08-00622-s001.zip › Figure S5.pdf]
