# Supplementary material for: Peroxins in Peroxisomal Receptor Export System Contribute to Development, Stress Response, and Virulence of Insect Pathogenic Fungus Beauveria bassiana
Source: J Fungi (Basel). 2022 Jun 10;8(6):622. doi: 10.3390/jof8060622 (PMC9224678; doi:10.3390/jof8060622)
Supplement: Supplementary file 1 [file jof-08-00622-s001.zip › Table S1.pdf]

Table S1 Primers used in this study.

Primer name	Sequence (5'-3')	Purpose
**PEX1**		
P_x1	GAGCTGTACAAGTAACCCGGGATAGGCTTTCTTGTGCTGG	Amplifying 5′-fragment
P_x2	TGGCTGCAGGTCGACGGATCCGCCCGTCCTTAGATGCTC	
P_x3	GACCCATGGCTCGAGTCTAGAATCGCACATACCATCAACAT	Amplifying 3′-fragment
P_x4	GGTGGTGGTGGCTAGCGTTAACGTCAACAGAACGGAAGAAAG	
P_x5	ATGATGGGCGGTTGTGAT	PCR for screening mutants
P_x6	GCTGCTGCTCTGAGTTTGC	
P_x7	ATCCGTCGACCTGCAGCCAAGCTTAACTGCTTGCCCTTCTTCT	Cloning the entire ORF and promoter
P_x8	ACACTAGTCAGATCTTCTAGTGTCCGAGTGACGGACTTTGA	
PL_x1	ATTCAATCACAAACACCTTCAAAATGGCTCCTAGAAGGAATGCGCAAT	Cloning coding sequence
PL_x2	CTCCTCGCCCTTGCTCACCATCATGAGACTACTACGGCCTCCAATC	
AD-Pex1-F	GCCATGGAGGCCAGTGAATTCATGGCTCCTAGAAGGAATGCG	For Y2H test
AD-Pex1-R	CAGCTCGAGCTCGATGGATCCCATGAGACTACTACGGCCTCCAAT	
**PEX6**		
P_x1	GAGCTGTACAAGTAACCCGGGTGCTTGTCGGAGATGTTG	Amplifying 5′-fragment
P_x2	TGGCTGCAGGTCGACGGATCCTACTGCTGGGTGAAATGG	
P_x3	AACGTCGACCCATGGCTCGAGCGAGACCGTCTTTGTGAG	Amplifying 3′-fragment
P_x4	ACACTAGTCAGATCTTCTAGACGAGGTTGGCAAGGAGCA	
P_x5	CTTGACAGTTCCACGCCTCT	PCR for screening mutants
P_x6	CGACTTGCTGAGTAGGGTGTTC	
P_x7	ATCCGTCGACCTGCAGCCAAGCTTCCGTGGAGCAGCAAGACT	Cloning the entire ORF and promoter

P_x8	ACACTAGTCAGATCTTCTAGTGTGAGGAGGATGAGGAGAAGAAGA	
PL_x1	ATTCAATCACAAACACCTTCAAAATGACGACAGCCTCGACCAGCGCGG	Cloning coding sequence
PL_x2	CTCCTCGCCCTTGCTCACCATATACAAATCCTCATCATCACTCGCC	
BD-Pex6-F	ATGGCCATGGAGGCCGAATTCATGACGACAGCCTCGACCAGCGCGG	For Y2H test
BD-Pex6-R	CGCTGCAGGTCGACGGATCCATACAAATCCTCATCATCACTCGCC	
AD-Pex6-F	GCCATGGAGGCCAGTGAATTCATGACGACAGCCTCGACCAGCGCGG	For Y2H test
AD-Pex6-R	CAGCTCGAGCTCGATGGATCCATACAAATCCTCATCATCACTCGCC	
**PEX26**		
P_x1	GAGCTGTACAAGTAACGCATTCCAACGACTTTC	Amplifying 5′-fragment
P_x2	CGACGGATCCCCGGGCTGCGGGTTTGCTATTCG	
P_x3	GACCCATGGCTCGAGTCTAGACTTGGATTTCGGAAACAGG	Amplifying 3′-fragment
P_x4	GGTGGTGGTGGCTAGCGTTAACAAAGGATCGTGACGGGTG	
P_x5	CACATCACCGCTTGTCTGC	PCR for screening mutants
P_x6	CGTAGTCCCACTCATTGTTGC	
P_x7	ATCCGTCGACCTGCAGCCAAGCTTGGAAATACCTGGACCCTAA	Cloning the entire ORF and promoter
P_x8	ACACTAGTCAGATCTTCTAGTGTCTAACCTGCCTCCCTCTAC	
PL_x1	ATTCAATCACAAACACCTTCAAAATGTCCTTTGATGGCTCCTACGCTC	Cloning coding sequence
PL_x2	CTCCTCGCCCTTGCTCACCATGATGTAGCTGACCTTGGTTCCCATG	
BD-Pex26-F	ATGGCCATGGAGGCCGAATTCATGTCCTTTGATGGCTCCTACGCTCCGT	For Y2H test
BD-Pex26-R	CGCTGCAGGTCGACGGATCCTTAGATGTAGCTGACCTTGGTTCCCATGC	
